# Hemp Seed Oil Inhibits the Adipogenicity of the Differentiation-Induced Human Mesenchymal Stem Cells through Suppressing the Cannabinoid Type 1 (CB1)

**DOI:** 10.3390/molecules29071568

**Published:** 2024-03-31

**Authors:** Albatul S. Almousa, Pandurangan Subash-Babu, Ibrahim O. Alanazi, Ali A. Alshatwi, Huda Alkhalaf, Eman Bahattab, Atheer Alsiyah, Mohammad Alzahrani

**Affiliations:** 1 Department of Food Science and Nutrition, College of Food Science and Agriculture, King Saud University, P.O. Box 2460, Riyadh 11451, Saudi Arabia; alshatwi@ksu.edu.sa; 2 Department of Human Nutrition, College of Home Economics, King Khalid University, P.O. Box 3236, Abha 10001, Saudi Arabia; 3 The Healthy Aging Research Institute, King Abdulaziz City for Science and Technology, P.O. Box 6086, Riyadh 11442, Saudi Arabia; iaenazi@kacst.edu.sa (I.O.A.); halkhalaf@kacst.edu.sa (H.A.); ebahattab@kacst.edu.sa (E.B.); 4 Genome Research Unit, Department of Biochemistry, College of Science, King Saud University, P.O. Box 2460, Riyadh 11451, Saudi Arabia; 5 The Applied Genomics Research Institute, King Abdulaziz City for Science and Technology, P.O. Box 6086, Riyadh 11442, Saudi Arabia; aalsayah@kacst.edu.sa; 6 Institute of Advanced Agricultural and Food Technologies, King Abdulaziz City for Science and Technology, P.O. Box 6086, Riyadh 11442, Saudi Arabia; mzahrani@kacst.edu.sa

**Keywords:** stem cells, cannabidiol, cannabinoid receptor, adipogenesis, lipogenic enzymes

## Abstract

Central and peripheral mechanisms of the endocannabinoid system (ECS) favor energy intake and storage. The ECS, especially cannabidiol (CBD) receptors, controls adipocyte differentiation (hyperplasia) and lipid accumulation (hypertrophy) in adipose tissue. In white adipose tissue, cannabidiol receptor 1 (CB1) stimulation increases lipogenesis and inhibits lipolysis; in brown adipose tissue, it decreases mitochondrial thermogenesis and biogenesis. This study compared the availability of phytocannabinoids [CBD and Δ9-tetrahydrocannabinol (THC)] and polyunsaturated fatty acids [omega 3 (ω3) and omega 6 (ω6)] in different hemp seed oils (HSO). The study also examined the effect of HSO on adipocyte lipid accumulation by suppressing cannabinoid receptors in adipogenesis-stimulated human mesenchymal stem cells (hMSCs). Most importantly, Oil-Red-O′ and Nile red tests showed that HSO induced adipogenic hMSC differentiation without differentiation agents. Additionally, HSO-treated cells showed increased peroxisome proliferator-activated receptor gamma (PPARγ) mRNA expression compared to controls (hMSC). HSO reduced PPARγ mRNA expression after differentiation media (DM) treatment. After treatment with HSO, DM-hMSCs had significantly lower CB1 mRNA and protein expressions than normal hMSCs. HSO treatment also decreased transient receptor potential vanilloid 1 (TRPV1), fatty acid amide hydrolase (FAAH), and monoacylglycerol lipase (MGL) mRNAs in hMSC and DM-hMSCs. HSO treatment significantly decreased CB1, CB2, TRPV1, and G-protein-coupled receptor 55 (GPCR55) protein levels in DM-hMSC compared to hMSC in western blot analysis. In this study, HSO initiated adipogenic differentiation in hMSC without DM, but it suppressed CB1 gene and protein expression, potentially decreasing adipocyte lipid accumulation and lipogenic enzymes.

## 1. Introduction

The endocannabinoid system (ECS) is a complicated cell signaling system that governs many aspects of the body’s physiology, including appetite, mood, pain, sensation, and immunological function. Recent developments shed light on how the ECS regulates the metabolism of adipose tissue. ECS influences various physiological processes related to adipocytes, such as phenotype determination and stimulating preadipocyte proliferation [1]. The primary components of this ECS included endocannabinoids, cannabinoid receptors and non-cannabinoid receptors [2].

The most well-known endocannabinoids are anandamide (AEA) and 2-arachidonoylglycerol (2-AG). Endocannabinoids are produced by the body basis of necessary, serving as signaling molecules that selectively bind to specific receptors. CB1 and CB2 receptors are the two main types of cannabinoids. The CB1 receptor is found primarily in the brain and central nervous system, whereas the CB2 receptor is most frequently located in the Immune system and other body tissues. The CB1 receptor was identified in adipocytes in 2003 marked the initial recognition of ECS specific role in adipocyte physiology [1]. In addition to CB1 and CB2, G-protein-coupled receptor 55 (GPCR55) and transient receptor potential vanilloid 1 (TRPV1) have been recently identified as non-cannabinoid receptors of the ECS [3,4]. Moreover, the ECS primary enzymes involved in this process are fatty acid amide hydrolase (FAAH), which breaks down anandamide, and monoacylglycerol lipase (MGL), which breaks down 2-AG [5].

There are naturally occurring substances that regulate the ECS. For example, the psychoactive Δ9-tetrahydrocannabinol (THC) derived from cannabis mimics endocannabinoid activity and activates ECS [3]. However, the non-psychoactive cannabidiol (CBD) blocks the CB_1_ receptor, leading to the downregulation of the ECS [6]. Furthermore, a high intake of omega 3 (ω3) downregulates ECS in adipose tissue, heart, and liver [7], while a high intake of omega 6 (ω6) upregulates ECS, suggesting an inverse relationship between ECS tone and ω3 levels and a positive relationship between ECS tone and ω6 levels in the body. Hemp seeds, derived from the *Cannabis sativa* L. plant, have been a crucial source of food, fiber, and medicine for centuries [8]. Hemp seeds contain elevated levels of the natural CBD and a trace level of THC [9]. Additionally, hemp seed contains major essential fatty acids, including ω3 and ω6, representing the optimal ratio of ω3:ω6 (1:3) [8].

Distinct dietary bioactive compounds can impact various stages of the cell cycle. Human mesenchymal stem cells (hMSCs) may experience differentiation to new cell types, including adipocytes, in response to natural products [10]. Hemp seeds represent most compounds that alter the ECS, including polyunsaturated fatty acids (ω3 and ω6), THC, and CBD. Nevertheless, the increasing recreational use of hemp seed makes comprehending how this compound affects the ECS balance essential. Therefore, we hypothesize that hemp seed oil may have a regulatory effect on the ECS, controlling adipogenesis, fat accumulation, and hMSC differentiation to the preadipocyte stage. This research will focus on different aspects of hemp seed oil (HSO). Initially, the hemp seed oil was extracted using shell (S) and no-shell (NS) seeds with different extraction methods, and the phytoconstituents were compared to cold-pressed oil. Furthermore, we studied its ability on adipogenic differentiation among hMSCs, and the involvement of the ECS system downstream gene and protein signaling cascade molecules expression levels have been analyzed. Our results may lead to the involvement of hemp seed and its derivatives in the alteration of the ECS system and its association with lipid metabolism during the early stages of adipocyte differentiation.

## 2. Results

### 2.1. Soxhlet Extraction Method Produces More Oil and Cold-Pressed Hemp Seed Oil Has the Optimal Ratios to Carry out the Research

Due to inconsistencies in the scientific literature regarding the chemical components of hemp seeds [11], we sought to test the products used in this study to establish a more coherent understanding of the seeds and select the optimal product for the research. To this end, HSO was extracted from shelled (S) and no-shell (NS) hemp seeds following three different extraction techniques [dynamic maceration (DyM), Soxhlet (Sox), and cannabinoids extracting method (CEM)] using methanol to chloroform (3:1) solution (10 g/experiment). Extracted oils were then compared to the ready-to-use cold-pressed hemp seed oil using GC–MS and HPLC to identify the composition of lipids and cannabinoids. Table 1 shows the oil yield of each extraction method and its average ± SD. Sox extraction had the most oil yield, with an average of 3.8 mL compared to 3 mL from DyM experiments and 2.75 mL from the CEM experiments. Also, it is well noted that Sox extraction from NS hemp seeds significantly yielded more oil than DyM and CEM, S hemp seeds extraction (*p*-value ≤ 0.002) (Table 1).

GC–MS results showed that Soxhlet (Sox) experiments yield the highest amount of ω3 and ω6 compared to DyM and CEM. In addition, long-chain saturated fatty acids (LCSFA), including palmitic acid and stearic acid, were present among all extraction methods; however, Sox extraction presented both acids. Meanwhile, DyM presented 1.79% stearic acid from S hemp seeds only. On the other hand, CEM presented palmitic acid only, and it was higher in S hemp seeds compared to NS hemp seeds. Additionally, CEM yields a remarkably high amount of ω6 (60.71%) compared to a meager amount of ω3 (0.695%). Regarding CBD and THC, they were not detected by GC–MS in all samples. Table 2 displays the compounds detected by GC–MS and their percentage.

HPLC results showed a trace amount of CBD but not THC in all samples. The highest amount of CBD was found in the cold-pressed HSO (0.327 ppm) compared to Sox (S) (0.162 ppm) and Sox (NS) (0.144 ppm). Table 3 displays the amount of CBD detected by HPLC and their concentration (ppm). GC–MS chromatograms of Sox extraction of NS hemp seeds, Sox extraction of S hemp seed oil, and HPLC chromatogram of HSO (Sox extraction) are presented in Figure S1. The observed results confirmed that cold-pressed techniques possess the favorable ratio of ω3 and ω6 fatty acid, and the availability of highest CBD compared to the Sox, DyM, and CEM methods of extraction. So, further studies were carried out using cold-pressed oil.

**Figure 1:**
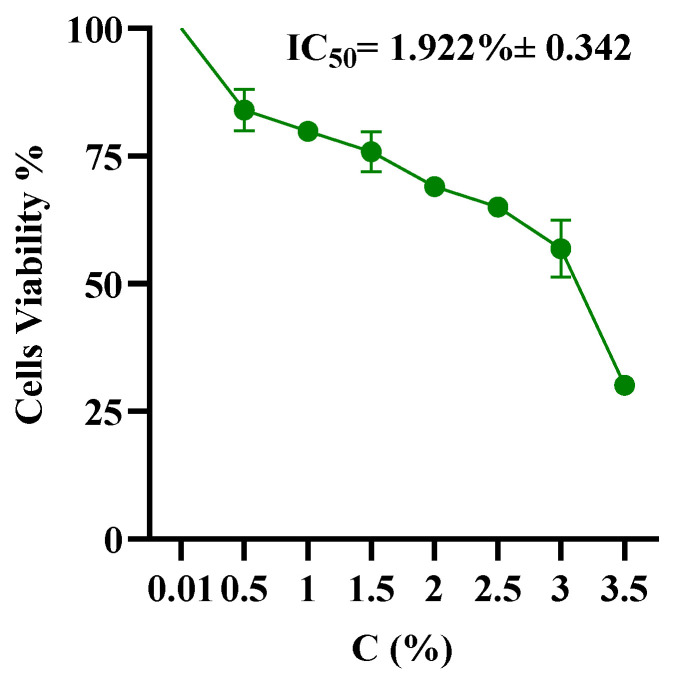
The IC_50_ of hMSC after 72 h incubation with HSO at various concentrations.

Next, to examine the cytotoxic effect of HSO on hMSC, we seeded hMSCs at 5 × 10^4^ cells/well in a 96-well plate and incubated for 24 h. HSO was emulsified using tween-20 at various concentrations (0.5%, 1%, 1.5%, 2%, 2.5%, 3%, 3.5%), then mixed with medium. Later, cells were treated with MTS in accordance with the directions given by the manufacturer after 72 h. A microplate reader was used to measure the absorbance at 570 nm. MTS assay indicated that the lower range of HSO was not toxic. A higher concentration of the HSO was required to kill the hMSCs. As shown in Figure 1, the IC_50_ value for HSO-treated cells was (1.922% ± 0.342). Ultimately, two physiobiological doses of HSO (0.1% and 0.05%) were carried out through all experiments.

### 2.2. HSO, CBD, and THC Differentiate hMSC into Adipocyte

**Figure 2:**
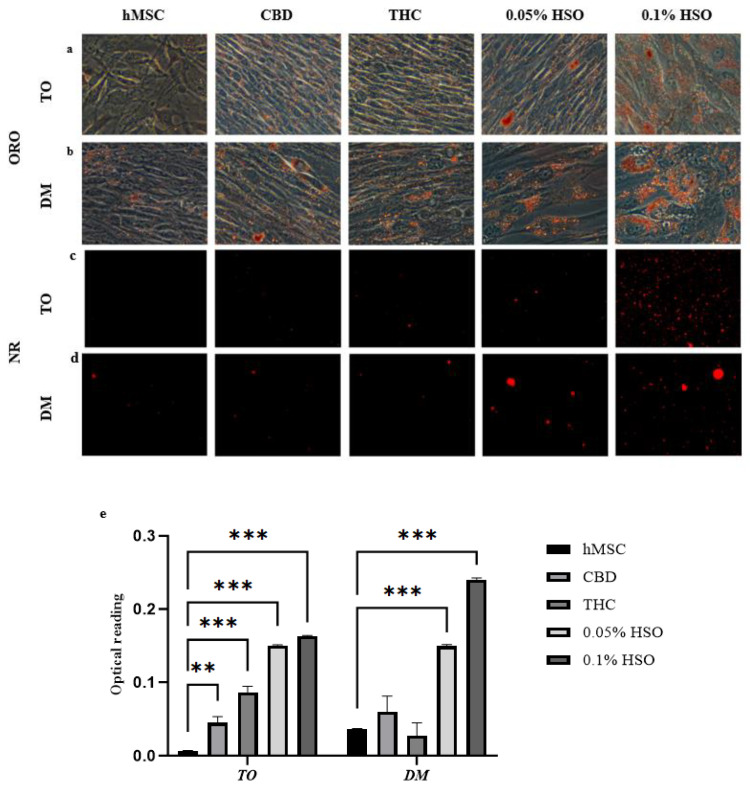
The morphological changes in hMSC after 72 h incubation with CBD, THC, 0.05% HSO, or 0.1% HSO in either Differentiation Media (DM) or Treatment Only (TO) using (**a**,**b**) ORO stain, (**c**,**d**) fluorescence dye—Nile Red at 50µm scale. (**e**) Intracellular lipid content represented by ORO density using 100% absolute isopropanol; the mean ± SD are shown; ANOVA as ** *p* ≤ 0.01, *** *p* ≤ 0.001.

Since active ECS favors adipogenesis, we thought to study the effect of two doses of HSO on hMSC and compare the results with CBD (ECS antagonists) and THC (ECS agonists) with and without differentiation media. Thus, hMSCs were seeded in 24-well plates and allowed to reach 70% confluence. Then, cells were treated with media containing treatment only (TO) or media containing differentiation agents (DM) + treatment for 72 h. Both the groups were treated with 0.05% HSO, 0.1% HSO, 1 µM of CBD, or 1 µM THC. To confirm the adipogenic differentiation among hMSCs, we first performed an Oil Red O (ORO) and Nile red staining. As appears in Figure 2a,b (ORO) and Figure 2c,d (NR), HSO (in a dose-dependent manner), CBD, and THC started the adipogenic differentiation on hMSC with or without DM. Subsequently, the intracellular lipid content was measured by quantifying the absorbance of the released Oil Red O bound at 620 nm after adding 400 μL of 100% isopropanol overnight. As seen in Figure 2e, the ORO bound test showed that all treatment groups were significantly higher in their lipid content compared to their controls (hMSC for TO and DM-hMSC for DM).

**Figure 3:**
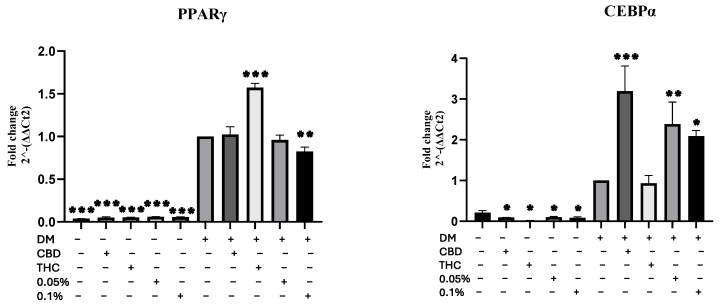
Gene expression. mRNA levels of adipogenic gene (PPARγ and CEBPα) were investigated by quantitative RT-PCR after 72 h of culturing in CBD, THC, 0.05% HSO, or 0.1% HSO treatments with/without DM; the mean ± SD are shown compared to the DM control; ANOVA as * *p* ≤ 0.05, ** *p* ≤ 0.01, *** *p* ≤ 0.001.

Second, we sought to confirm the Oil Red O by quantifying the transcription levels of fat accumulation and adipogenic genes: peroxisome proliferator-activated receptor gamma (PPARγ) and CCAAT/enhancer-binding protein alpha (CEBPα). Subsequently, cells were harvested, cDNA was prepared and utilized for quantitative reverse transcription-polymerase chain reaction (qRT-PCR) analysis. Results showed that expression levels of these genes differed significantly between TO and DM (Figure 3). As far as PPARγ expression is concerned, Figure S1 shows the 2^−ΔΔCt2^ (fold change) between each group compared to the control group (hMSC). Although there was a very minimal increase among all TO groups, it was significantly higher than the control group when compared with DM groups (Figure S2). However, because the fold change of the TO groups was minimal, whereas the fold change of the DM groups was enormous in comparison to the control (hMSC), we decided to compare the results to the DM control to determine whether adding the treatments to the DM affected the results significantly. As Figure 3 shows, adding THC to the DM has increased PPARγ expression significantly by 0.573-fold ± 0.132 compared to the DM control (*p* ≤ 0.001); meanwhile, adding 0.1% HSO treatment to the DM has decreased PPARγ expression significantly by 0.177-fold ± 0.130 compared to the DM control (*p* ≤ 0.01). Similarly, the fold change of the CEBPα mRNA expression among DM groups was higher than TO when compared to the control hMSCs (Figure S1). Also, comparing all groups to the DM control showed that all DM treatments except THC have significantly increased the expression of CEBPα mRNA. Meanwhile, a significant decrease was seen among all TO groups (*p* ≤ 0.05) (Figure 3). Interestingly, Figure S2 shows that comparing the groups (TO and DM) separately from each other resulted in the following: among the TO groups, all treatments groups (CBD, THC, 0.05% HSO and 0.1% HSO) have decreased the expression of CEBPα mRNA significantly, and the most decrease was observed among the THC treatment group by 0.9095-fold ± 0.345 (*p* ≤ 0.001). However, among the DM groups, adding treatments to the DM has increased the expression of CEBPα mRNA except for the THC treatment that decreased the expression, suggesting that THC might have a direct effect on the expression of CEBPα mRNA (Figure S2).

### 2.3. HSO, CBD, and THC Treatments Regulated the ECS

**Figure 4:**
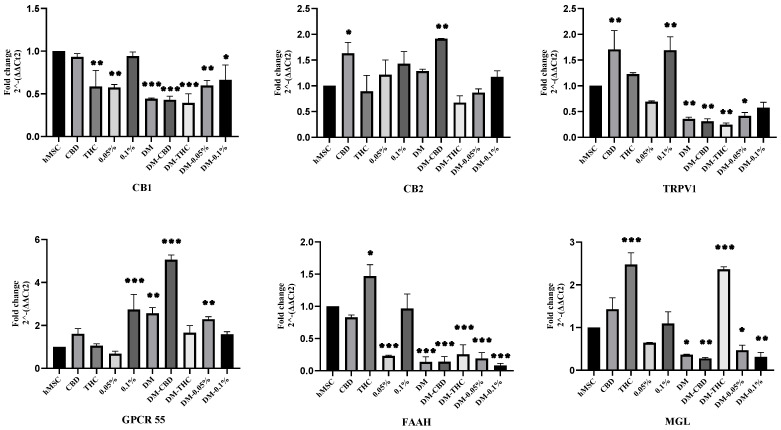
Gene expression. mRNA levels of ECS genes (CB1, CB2, TRPV1, GPCR55, FAAH, and MGL) were investigated by quantitative RT-PCR after 72 h of culturing with CBD, THC, 0.05% HSO, or 0.1% HSO treatment with/out DM; the mean ± SD are shown; ANOVA compared to hMSC as * *p* ≤ 0.05, ** *p* ≤ 0.01, *** *p* ≤ 0.001.

Since HSO and its active compounds (CBD and THC) initiated the adipogenic differentiation, we sought to examine whether this effect was related to the activation of the ECS. Thus, we studied the trends of EC receptors (CB1, CB2, TRPV1, GPCR 55) and degrading enzymes (FAAH and MGL) in terms of their mRNAs expression upon treatment with TO or DM. As shown in Figure 4, CB1 mRNA expression compared to the control (hMSC) was significantly downregulated in all groups except for TO-CBD, and TO-0.1% HSO was decreased but not significantly. Meanwhile, CB2 mRNA expression was significantly upregulated upon the treatment with CBD in both TO and DM groups by 0.631 ± 0.212 (*p* ≤ 0.05) and 0.915 ± 0.008 (*p* ≤ 0.01), respectively. It is possible that CBD is a direct activator of CB2. On the other hand, THC treatment has decreased the CB2 mRNA expression in both groups (TO and DM) but not significantly. Generally, a similar pattern can be noted in the CB2 mRNA expression when comparing the TO groups to the DM groups (Figure 4). Regarding TRPV1 mRNA expression, we found that adding DM has downregulated the expression significantly in all DM groups, except DM-0.1% HSO was not significant. However, TO-0.1% HSO along with TO-CBD has significantly increased the expression of TRPV1 mRNA (*p* ≤ 0.01). In terms of GPCR55 mRNA, adding DM has increased the GPCR55 mRNA expression in all DM groups but was only significant among DM control, DM-CBD, and DM-0.05% HSO (*p* ≤ 0.01, *p ≤* 0.001, and *p ≤* 0.01, respectively). Interestingly, TO-CBD has also increased the expression of GPCR55 mRNA but not significantly, whereas TO-0.1% HSO has significantly increased the expression of GPCR55 mRNA (*p* ≤ 0.001). As far as the EC enzymes FAAH and MGL are concerned, we found that adding DM has significantly decreased FAAH mRNA in all groups (*p* ≤ 0.001) and significantly decreased MGL mRNA in all groups except for the DM-THC group, where it was significantly increased (*p* ≤ 0.001). Interestingly, THC treatment has significantly increased both enzymes compared to their controls. THC treatment has increased the expression of MGL mRNA significantly (*p* ≤ 0.001) in the TO group. Furthermore, THC treatment in the TO has increased the expression of FAAH mRNA significantly (*p* ≤ 0.05), suggesting that there might be a direct relationship between THC treatment and EC-degrading enzymes (FAAH and MGL).

**Figure 5:**
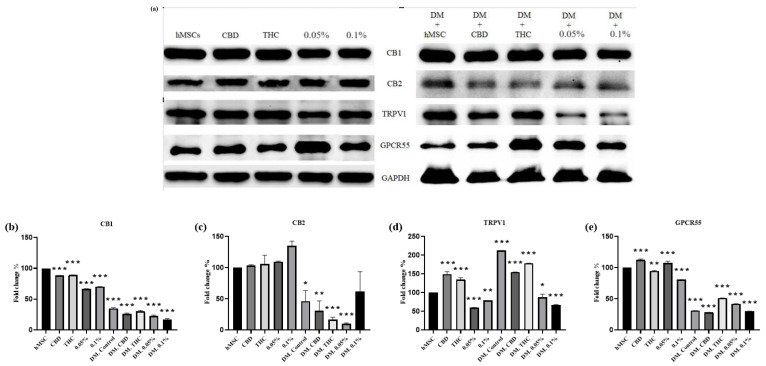
Western blotting of ECS receptors on hMSCs and treatments with or without DM (**a**). Densitometry analysis of CB1, CB2, TRPV1, and GPCR55 are shown in (**b**–**e**), respectively. The means ± SD are shown; ANOVA as * *p* ≤ 0.05, ** *p* ≤ 0.01, *** *p* ≤ 0.001.

Next, we sought to study the protein expression trends of the EC receptors (CB1, CB2, TRPV1, GPCR55). To this end, cells were harvested, and immunoblotting was performed on whole-cell lysates followed by blot quantification using densitometry scanning. As Figure 4 shows, protein expression of CB1 has confirmed the CB1 mRNA expression and was significantly decreased in all groups (*p* ≤ 0.001), and the highest decrease was among the DM-treated groups. As far as CB2 protein expression is concerned, it was significantly decreased in all DM-treated groups, except DM-0.1% HSO was not significant; meanwhile, it was slightly but not significantly increased in all TO groups. It is noteworthy that CB2 protein expression was upregulated upon the treatment of 0.1% HSO when comparing each treated group to their controls. Figure 5d shows that TRPV1 protein expression has upregulated in the CBD- and THC-treated groups in the TO and DM; meanwhile, it was downregulated after the oil treatment (0.05% HSO and 0.1% HSO) in both TO and DM groups. In contrast to the mRNA result, we found that the protein level of TRPV1 in DM, DM-CBD, and DM-THC was significantly higher than the control (*p* ≤ 0.001). This could be due to the stableness and degradation resistance of the resulting protein even with low levels of mRNA. In addition, some proteins have longer half-lives than their mRNA, despite the rapid degradation of their mRNA [12]. As far as GPCR 55 protein levels are concerned, Figure 5e shows that the expression was notably and significantly decreased in all DM-treated groups compared to the control (*p* ≤ 0.001). However, the expression of GPCR55 in DM-THC, DM-0.05% HSO, and DM-0.1% HSO was increased compared to their control (DM control). In addition, GPCR 55 protein expression was significantly increased in the TO-0.05% HSO and TO-CBD (*p* ≤ 0.001).

## 3. Discussion

In this investigation, we aimed to identify the role of active ECS in the effects of HSO on adipogenesis in hMSCs. Cold-pressed HSO used in this study contained around a 0.5:1 ratio of ω3:ω6 and 0.327 ppm CBD. CBD (1 µM) and THC (1 µM) treatments were used for comparison. Our present study confirmed for the first time that HSO was able to initiate adipocyte differentiation among hMSCs without the differentiation media (DM) in a dose-dependent manner (Figure 2). Additionally, HSO treatment increased the size of adipocytes in DM-treated cells (Figure 2). This might be due to the high lipid accumulation in DM-treated cells. Similarly, based on ORO results in Figure 2, we found that CBD and THC treatments in TO and DM have initiated the adipogenic differentiation compared to the control (hMSC). However, THC treatment in the DM-treated cells have decreased intracellular lipid content compared to its control (DM control) (Figure 2e). This could be attributed to the fact that the acid form of THC (THCA) creates fewer and smaller lipid droplets than rosiglitazone in hMSCs [13,14] and, as previously stated, that most of the CBD and THC were appropriately stored in hemp seed oil in the form of acidic precursor [15,16]. Furthermore, as PPAR-γ and CEBP-α are two master transcription factors that govern the differentiation and proliferation of adipocytes [17], we confirmed that the adipogenic gene CEBP-α expression level has been significantly downregulated in HSO-, CBD-, and THC-treated hMSCs compared to its control (hMSC) (Figure S2a); however, adding DM increased the CEBP-α gene expression significantly in HSO- and CBD-treated maturing adipocytes (DM-cells) compared to its control (DM-hMSC) (Figure S2b). Interestingly, we found that THC treatment has downregulated the expression of the CEBP-α mRNA in both TO- and DM-treated cells (Figure S2), suggesting a direct relationship between THC treatment and CEBP-α mRNA expression. However, THC treatment has significantly increased the expression of PPAR-γ mRNA in both TO- and DM-treated cells when compared to the control in each group (Figure S2). It is also noteworthy that all treatments in TO (HSO, CBD, and THC) have increased the expression of PPAR-γ mRNA compared to their control (hMSC). However, in the DM-treated cells, HSO treatment has decreased the PPAR-γ mRNA expression compared to the DM control (Figure S2). Both HSO treatments (0.1% HSO and 0.05% HSO) in the DM-treated cells have decreased the expression of PPAR-γ mRNA—in a dose-dependent manner—by 0.04-fold ± 0.053 and 0.176-fold ± 0.057, respectively (Table S2). This suggests that HSO might have a controlling role of lipid accumulation and adipogenesis. In addition, the ability of CBD administration to activate PPAR-γ mRNA and drive adipogenesis in both mice and hMSCs has been validated and identified such that CBD’s impact might be attributed to its tendency to stimulate lipogenesis without the balance of lipolysis [18]. CBD in our study has similarly increased the expression of PPAR-γ mRNA significantly (*p* > 0.05) when in hMSCs; however, adding CBD to DM has maintained the same expression levels compared to the DM control.

We also studied the EC receptors’ and enzymes’ involvement in hMSC adipocyte differentiation with and without differentiation media. Upon discovery of cannabinoid receptor-1 (CB1) and its role in peripheral organs, many studies led to the discovery that CB1 receptors are directly involved in the physiological processes of energy homeostasis in adipocytes and many tissues [19,20]. CB1 receptor agonist in adipocytes and chronic activation in white adipose tissue have been found with impaired mitochondrial functions and decreased mitochondrial biogenesis [20]. In contrast, Roche et al. [19] found that activation of the CB1 receptor with the endocannabinoid 2-AG has increased the intracytoplasmic cyclic adenosine monophosphate (cAMP), which may lead to increased lipolysis and decreased lipid accumulation. Another study found that CB1 antagonist stimulates the expression of mitochondrial biogenesis enzymes, including Krebs cycle, and increased the oxidative phosphorylation potential [20]. In the current study, all treatments—especially HSO—significantly decreased CB1 mRNA expression and protein expression in all groups (*p* ≤ 0.001); meanwhile, DM-treated cells showing the highest reduction. The adipogenic effect of HSO on hMSCs is evident by ORO and PPAR-γ mRNA expression—in an early stage—but might not be mediated by EC CB1 receptor. Bajzer et al. [21] confirmed that persistent CB1 antagonism has been linked to increased energy consumption, lipolysis, mitochondrial biogenesis, and activation of thermogenesis. Further, inhibition of adipocyte CB1 potentially influences lipid homeostasis in a positive way and the production of adipokines, which facilitate the lipolysis or inhibit lipid accumulation and hypertrophy [1,22].

As far as cannabinoid receptor-2 (CB2) is concerned, it was previously linked to immune system cells only. However, Roche et al. [19] were able to discover the biological functions of CB2 receptor in human pre-adipocytes and mature adipose tissue; the activation of adipocyte CB2 receptor inhibits cyclic adenosine monophosphate (cAMP) synthesis, which may lead to decreased lipolysis. In the present study, we found that CBD treatment showed strong affinity towards CB2 compared to THC. CBD treatment has significantly increased the CB2 mRNA expression in both TO- and DM-treated cells (*p* ≤ 0.05, *p ≤* 0.01, respectively). On the other hand, THC treatment has decreased the CB2 mRNA expression in both TO- and DM-treated cells (not significantly). Furthermore, the CB2 protein levels have been slightly elevated in both CBD and THC treatments in the TO; however, when added to DM-treated cells, both treatments have decreased the CB2 protein levels significantly (*p* ≤ 0.01, *p ≤* 0.001, respectively). Meanwhile, HSO treatment, especially 0.1% HSO, has slightly elevated the expression of CB2 mRNA in both TO- and DM-treated cells, and held this elevation even at protein level, suggesting that there are other substances in HSO that activate the CB2 receptor besides CBD, such as arachidonic acid, an ω6 PUFA that is a precursor for AEA.

Another EC receptor is TRPV1; AEA and other endocannabinoids are natural ligands for TRPV1 [23,24], and it is produced when intracellular Ca^2+^ levels increase [3]. In addition, a cross talk between CB1 and TRPV1 has been demonstrated by several studies [25,26,27,28]. CB1 has been demonstrated to either enhance or reduce TRPV1 channel activity [26]. In the present study, we found similar expression trends between TRPV1 and CB1 (Figure 4), as CBD and 0.1% HSO held the highest expression of CB1 compared to other treatment groups, it also increased the expression of TRPV1 mRNA significantly (*p* ≤ 0.01). Similarly, as DM has decreased the expression of CB1 in all treatment groups significantly, it also decreased the TRPV1 mRNA expression significantly, except for 0.1% HSO it was not significant. However, this was not the case when it comes to TRPV1 protein expression, where DM-treated cells (DM control), DM-CBD, and DM-THC have significantly increased the TRPV1 protein expression. Meanwhile, HSO treatments (0.05% HSO and 0.1% HSO) have decreased its expression significantly in both TO and DM. It appears that the interaction between CB1 and TRPV1 is dependent on the amount of circulating AEA. Research has shown that TRPV1 may be activated by supplementing high dosages of AEA (1–10 M) [28,29]. In contrast, low doses of AEA (3–30 nM) decrease TRPV1 expression [30,31]. Therefore, the enzymatic production and breakdown of endogenous cannabinoids may be crucial determinants of TRPV1 activity in tissues that co-express TRPV1 and CB1 [25].

The enzymes FAAH and MGL are degrading enzymes of the intracellular endocannabinoids AEA and 2-AG, produced in response to high levels of the endocannabinoid to retain hemostasis of the active ECS [20,32]. In the present study, we found that FAAH mRNA expression was downregulated in all groups except TO-THC treatment. Significant reduction of FAAH mRNA was noted among all DM-treated groups (*p* ≤ 0.001). Similar findings were observed on MGL mRNA expression. DM-treated groups have significantly reduced its expression except DM-THC, which significantly increased the expression of MGL mRNA (*p* ≤ 0.001). Also, THC treatment in the TO has significantly upregulated both FAAH and MGL mRNAs (*p* ≤ 0.05, *p ≤* 0.001, respectively), while CBD and HSO treatments have upregulated MGL mRNA and downregulated FAAH mRNA expression but not significantly. Furthermore, since FAAH is expressed on demand upon high levels of anandamide to retain homeostasis of the active ECS [33], our present study suggests that the ECS might not be highly activated in the differentiation process of hMSC to adipocyte. Also, the balance ratio of ω3 to ω6 in the HSO might have a role in maintaining lower endocannabinoids (AEA and 2-AG) and consequently lower the expression levels of FAAH and MGL, which are associated with low lipid accumulation. In this context, an in vitro study has demonstrated that ω3 polyunsaturated fatty acids (PUFA) have the potential to reduce the amounts of AEA and 2-AG in differentiated adipocytes from mice [34]. Conversely, arachidonic acid (ω6, PUFA) has been seen to elevate endocannabinoid levels [35].

l-α-lysophosphatidylinositol (LPI) is known as a regulator of the intracellular Ca^2+^ accumulation, and it is believed to be the only endogenous ligand of G-protein-coupled receptor-55 (GPCR55) that is presumed to be a cannabinoid receptor [36]. In addition, a positive association between obesity, type 2 diabetes, and GPCR55 expression in visceral adipose tissue (VAT) has been identified [37]. Consistently, obese patients have been identified with higher plasma levels of LPI than lean subjects [36]. In our study, we observed that all groups subjected to DM treatment exhibited a notable upregulation in the expression of GPCR55 mRNA. However, a contrasting trend was observed in the levels of GPCR55 protein, as DM treatment resulted in a significant decrease in its expression across all groups (*p* ≤ 0.001). Similar findings were noted in TO-HSO (both doses 0.05% HSO and 0.1% HSO), where TO-0.1% HSO has significantly increased GPCR55 mRNA but significantly reduced the protein levels, whereas TO-0.05% HSO decreased GPCR55 mRNA but significantly increased its protein expression.

Generally, the observed discrepancy between the findings of our research and those of previous studies might perhaps be attributed to variances in the level of maturation or cell type. In our current study, endpoint measures were conducted three days after initiating adipocyte differentiation in hMSCs and treatment, whereas other investigations employed distinct cell types or conducted in vivo experiments, or achieved a fully developed state of adipocyte, often after a differentiation period of 14 days.

## 4. Materials and Methods

### 4.1. Plant Products and Chemicals

Raw hemp seeds (shelled-S and not shelled-NS-Canadian source) and cold-pressed hemp seed oil (HSO) (Moreno Valley, CA, USA) were bought from iHerb (Irvine, CA, USA). Liquid Cannabidiol (CBD) and Δ^9^-tetrahydrocannabinol (THC) standards were obtained from RESTEK^®^ (Bellefonte, PA, USA). Powder CBD was obtained from Abcam (ab120448) (Cambridge, UK). Methanol, acetonitrile, chloroform, formic acid, acetone, and isopropanol HPLC grade were supplied thankfully by the King Saud University (KSU) chemical warehouse.

### 4.2. Instrument

Gas Chromatography–Mass Spectrometry (GC–MS) was utilized for the analysis of extracts to determine the chemical components present and their respective concentrations. The column utilized was a DB-5MS with dimensions of 30 m in length, 0.250 mm in diameter, and 0.25 μm in thickness. The flow rate was 3 mL/min, pressure set at 10.42 psi, operating in splitless mode, with an injection volume of 3 μL. The oven temperature was 60 °C for 3 min, then increased to 100 °C at a rate of 3 °C per minute, followed by reaching 200 °C in 1 min at the same rate, and finally reaching 300 °C in 1 min at a rate of 5 °C per minute. GC–MS data were interpreted using FAME and NIST libraries. The chromatogram threshold in all experiments was set at 17.

High-Performance Liquid Chromatography: Ultraviolet/Diode Array Detector (HPLC-UV/DAD): HPLC-UV/DAD (Shimadzu, Colombia, MD, USA) was used to identify and quantify chemical compounds using a standard for each analyte (CBD and THC). Hypersil^®^ column (100 × 4.6 mm, Thermo Scientific, Waltham, MA, USA) with a particle size of 5 µm ODS was used. The temperature was set at 30 °C, and injection volume was 10 microliters. Mobile phase solutions were A: 70% acetonitrile and B: 30% formic acid (0.1%). Samples and standards were prepared following Brighenti et al. [38]. The ratio of samples to isopropanol was 20 μL:4 mL, then vortexed for 30 s, and 2 mL was taken into an HPLC vial.

### 4.3. Extraction Method

Three extraction techniques (dynamic maceration-DyM, Soxhlet-Sox, and cannabinoids extraction method-CEM) were followed using methanol/chloroform solution (M/C, 3:1). DyM extraction: a modified method of Brighenti et al. [38] was followed, shelled-(S) and no-shell-(NS) hemp seeds (HS) were grounded for 30 s each. An amount of 10 g of extract(s) as initial weight was added with 40 mL of the solvent and placed on a magnetic stirrer for 4 h at room temperature. The solution was filtered using No. 1 Whatman™ filter papers (Maidstone, UK, 125 mm), and the residue was extracted twice with another 40 mL of the solvent, followed by 20 mL for 1 h 30 min and 30 min, respectively, and was placed on magnetic stirring. Finally, the filtrate was evaporated under a vacuum, and the crude extract was collected. Soxhlet-Sox extraction: a modified method of Devi and Khanam [39] was followed. An amount of 10 g of S or NS hemp seed powder was weighed and placed in a thimble (Whatman™ cellulose extraction thimbles 25 mm × 100 mm). Then, 100 mL of solvent was added to keep the solvent and HS ratio at 1:10 g/mL. The procedure was carried out for 6 h, the mixture was evaporated under a vacuum, and the crude extract was collected. CEM extraction: a modified method of Mandrioli et al. [40] was followed, whereby 10 g of S or NS hemp seed powder was mixed with 100 mL of solvent. The oscillator node was placed inside the beaker touching solvent (Physical Field) at 350 hertz and amplitude of 10 vpp and “Sin” wave shape for 30 min. Then, the beaker was placed in a sonic bath for 30 min. The process was repeated on the oscillator for 20 min and a sonic bath for 20 min. The mixture was then filtered into the evaporator flask using No. 1 Whatman™ filter papers (125 mm). Then, the mixture evaporated, and crude oil was collected. All extraction methods were performed in triplicate.

An evaporator was used at 55 °C throughout the experiments, along with a vacuum to collect the solvent-free crude extract. Then, crude extracts were collected in centrifuge tubes for further purification process. Firstly, liquid–liquid extraction (LLE) was utilized, 2 mL of crude extract was placed in a centrifuge tube along with 2 mL of H_2_O and 2 mL of methanol, centrifuged at 5000 rpm for 5 min, then 1.5 mL of the solvent layer was taken into an Eppendorf tube. Secondly, a syringe filter (PTFE-0.45 µm- Thermo Scientific, Waltham, MA, USA) was used, followed by another syringe filter (Nylon-0.45 µm- Thermo Scientific, Waltham, MA, USA), then brought to dryness using a stream of nitrogen. Finally, the residue was diluted in acetone for analysis.

### 4.4. Cell Culture

The hMSCs were obtained from the American Type Culture Collections (ATCC) in Manassas, VA, USA. Early passage of hMSCs (≤passage 3) were cultured in Dulbecco’s Modified Eagle’s Medium (DMEM, Gibco, Waltham, MA, USA). The cells were cultured in a medium containing 10% fetal bovine serum (FBS, Gibco™, Grand Island, NY, USA) and 1% penicillin–streptomycin (Gibco™, NY, USA) at 37 °C in a humidified environment with 5% CO_2_. At 70% confluency, cells were maintained in either DMEM + treatment only (5 groups: control/DMEM only, CBD, THC, 0.05% HSO, and 0.1% HSO for 72 h or in a differentiation media containing Dexamethasone (1 µM), IBMX (0.5 µM), Rosiglitazone (1 µM), recombinant human insulin (167 nM) for 72 h [41] along with treatments (5 groups: DM-hMSC, DM-CBD, DM-THC, DM-0.05% HSO, and DM-0.1% HSO) for 72 h. HSO was dissolved in 1% tween-20 to ensure homogenization. CBD and THC were dissolved in methanol. Finally, condition media of DM groups were collected for further use.

### 4.5. Cell Viability Assay

Human mesenchymal stem cells (hMSCs) were placed in a 96-well plate with a concentration of 1 × 10^4^ cells per well and left to incubate for 24 h. Cells were exposed to varying amounts of hemp seed oil (HSO) (0.5%, 1%, 1.5%, 2%, 2.5%, and 3%) for 72 h. HSO was dissolved in 1% tween-20 and emulsified using a sonication device at 30 °C and 40 RPM for 5 min to ensure homogenization. hMSC treated with 0.5% tween-20 was used as a control. Then, cells were treated with MTS in accordance with the directions given by the manufacturer after 72 h. A microplate reader (SPECTROstar^®^ Nano—BMG Labtech, Ortenberg, Germany) was used to measure the absorbance at 570 nm. Three replicate wells were used for each data point throughout the experiments. Ultimately, two physiobiological doses of HSO (0.05%) and (0.1%) were used along with (1 µM) of CBD and THC for comparison.

### 4.6. Oil Red O and Nile Red Staining

hMSC were seeded in a 24-well plate and allowed to reach 70% confluence. Cells were treated with HSO, CBD, and THC. After 72 h of treatment, growth media was removed, and cells were gently washed using phosphate-buffered saline (PBS). Subsequently, the cells were fixed with a 10% formalin solution for a duration of 1 h at room temperature, then washed three times with deionized water. Oil Red O (ORO) solution (4:6 water to 6% Oil Red O dye in isopropanol) was applied to the cells for 10 min, then unbound Oil Red O was removed by washing with PBS. The stained lipid droplets were captured using a light microscope (ZEISS, Axiocam 506 color, Jena, Germany). Finally, bound ORO were extracted with 100% isopropanol (400 μL/well) overnight, and the concentration of intracellular ORO was assessed, which is directly proportional to lipid accumulation. The absorbance at 620 nm was measured in duplicate wells using a microplate reader.

For Nile Red (NR) staining, media from the experimental cells were discarded and washed with PBS, further NR staining was added to each well. Then, the cells were incubated at room temperature for 10 min, then washed with deionized water to remove the unbound dye before imaging. The stained lipid droplets were captured using the green filter of an inverted florescence microscope (ZEISS, Axiocam 506 color).

### 4.7. RNA Extraction and Real-Time Polymerase Chain Reaction (RT-PCR)

hMSCs were seeded in a 24-well plate and allowed to reach 70% confluence. Cells were treated with HSO, CBD, and THC for 72 h; then, total RNA was extracted from hMSC using Qiagen, RNEASY MINI KIT (Qiagen, Inc., Tulsa, OK, USA). After that, the concentration and purity of the extracted RNA were measured using Quickdrop (SpectraMax, QuickDrop, Framingham, MA, USA). Then, 1 µg RNA was converted to cDNA using a High-Capacity cDNA Reverse Transcription Kit (Thermo Fisher Scientific, Vilnius, Lithuania). The QuantStudio 3 (Thermo Fisher Scientific, Waltham, MA, USA), along with SYBR^®^ green Universal Master Mix (Life Technologies Ltd., Paisley, UK), were used to quantify the transcription levels of specific genes (PPARγ, CEBPα, CB1, CB2, TRPV1, GPCR55, FAAH, MGL). For each sample, PCR reactions were repeated twice, and the transcription levels of each gene were standardized to GAPDH (primer sequences are presented in Table S3).

### 4.8. Immunoblot Analysis

Cell lysates were prepared using RIPA lysis buffer and Halt™ Protease inhibitor (PI) (Thermo Fisher Scientific, USA) at a 1:25 ratio (PI:RIPA). Cell lysates were homogenized for an hour on ice, then centrifuged at 14,000 rpm for 30 min at 4 °C. The Bradford assay (Bio-Rad Protein Assay kit, Hercules, CA, USA) was used to measure protein concentration. A total protein concentration was set at 35 µg/μL for all samples. The sample buffer was prepared at a 1:19 ratio of β-mercaptoethanol:2x-laemmli-sample-buffer. Then, a 1:1 ratio of samples to sample buffer was mixed and heated at 95 °C for 5 min before uploading to the 10% SDS-polyacrylamide gel electrophoresis (PAGE). Gels were transferred to 0.2 µm polyvinylidene difluoride blotting membrane (PVDF, Amersham™ Hybond™, Cytiva, München, Germany) after electrophoresis, then blocked for 1 h with 5% skimmed milk. Next, TBST 1X buffer (50 mL of TBS 20X + 950 mL DW + 1 mL of Tween 20) was used to rinse the membrane three times. Then, the membrane was incubated overnight at 4 °C with primary monoclonal antibodies anti-CB_1_ (rabbit, 1:4000), primary polyclonal antibodies anti-CB_2_ (rabbit, 1:200), primary polyclonal antibodies anti-TRPV_1_ (rabbit, 1:500), and primary polyclonal antibodies anti-GPCR55 (rabbit, 1:166) (Abcam). After that, the membrane was incubated for 2 h at room temperature with primary monoclonal antibodies anti-GAPDH (mouse, 1:10,000). The membrane was washed three times and then incubated for 1 h with anti-(rabbit/mouse) IgG secondary antibody horseradish peroxidase-conjugated (1:1000, Abcam) in 5% skim milk.

Finally, images were taken using the ChemiDoc™ Touch Imaging System (Bio-Rad, USA) after incubating the membrane in the Clarity Western ECL Substrate kit (Bio-Rad, USA). Image lab 6.1 software was used to quantify band intensities.

### 4.9. Statistical Analysis

The mean and standard deviation (SD) of at least duplicate experiments are used to represent all data. In addition, one-way ANOVA was used to examine the statistical significance among multiple groups, followed by the Dunnett test for multiple comparisons using GraphPad Prism 9.4.1 (Windows, GraphPad Software, San Diego, CA, USA) https://www.graphpad.com (accessed on 13 September 2022). Statistical significances were indicated as *p*-values ≤ 0.05.

## 5. Conclusions

The endocannabinoid system (ECS) is essential in promoting fat storage in animals through many mechanisms. EC tone in hMSC—including receptors and enzymes—was investigated following treatment with HSO (0.05% HSO and 0.1% HSO), CBD, and THC with and without DM during the early stages of adipogenesis. The agonism and antagonism of CB1 pose a direct effect on adipocytes and regulate energy intake and expenditure through central and peripheral pathways. In the present study, HSO effectively downregulated the CB1 mRNA, and protein expression further reduced the endogenous ligand TRPV1. Endogenous CB1 inhibition neutralizes or reduced the FAAH and MGL expressions, which play a major role in the endocannabinoid AEA and AA degradation. Although HSO independently has the capacity to initiate the adipogenic differentiation among hMSCs and upregulation of adipogenic gene PPAR-γ, HSO inhibits CB1, TRPV1, and PPAR-γ when differentiation was induced by DM. That indicates that HSO alone might have the ability to initiate the differentiation; however, it reduces the lipid accumulation and energy metabolism when differentiation is induced by DM compared to CBD or THC alone. Therefore, hemp seed might be considered as a natural agent for CB1 inhibition, to overcome adipocyte lipogenesis and obesity. Finally, we recommend an in vivo study to confirm whether HSO will continue to inhibit CB1 and define the adipocyte maturation.

## Figures and Tables

**Table 1:** Hemp seed oil yield following different extraction methods.

Type of Seed	Extraction Method	Average (mL)	Average (mL) by Extraction Method
S	DyM	2.4 ± 0.39	DyM
	Sox	3.3 ± 0.50	3 mL
	CEM	2.5 ± 0.25	Sox
NS	DyM	3.6 ± 0.52 ^d^	3.8 mL
	Sox	4.3 ± 0.19 ^a,b,c^	CEM
	CEM	3 ± 0.12	2.75 mL

Oil was extracted from 10 g of seeds. Each value is mean ± SD Sox (NS): ^a^—*p* = 0.002 compared to DM (S), ^b^—*p* = 0.002 compared to CEM (S), ^c^—*p* = 0.024 compared to CEM (NS). DM (NS): ^d^—*p* = 0.038 compared to DM (S). DyM: dynamic maceration; Sox: Soxhlet; CEM: cannabinoid extraction method; S: shilled hemp seed; NS: not-shilled hemp seed.

**Table 2:** Hemp seed extracted oil GC–MS analysis.

Oil Content	DyM		Sox		CEM		HSO
	S	NS	S	NS	S	NS	Cold-Pressed
ω3	30.65%	14.46%	28.78%	34.08% *	1.24%	0.69%	13.36%
ω6	41.98%	20.4%	64.74% *	55.02%	54.06%	60.71%	25.09%
Vitamin E	0	0	0	0	0	2.65% *	0
Palmitic acid	0	0	4.13%	6.78%	7.68% *	5.71%	5.02%
Stearic acid	1.79%	0	0.89%	2.20% *	0	0	1.87%
Pentadecanoic acid	0	0	0	0	0	0	3.80% *

* The highest among all extraction methods.

**Table 3:** CBD concentration in hemp seed extracted oils using HPLC.

DyM		Sox		CEM		HSO
S	NS	S	NS	S	NS	Cold-Pressed
0.131	0.141	0.162	0.144	0.101	0.109	0.327 *

* The highest among all extraction methods.

## Data Availability

The data presented in this study are openly available in FigShare at [https://doi.org/10.6084/m9.figshare.25218842.v1 (accessed on 1 March 2024)].

## Supplementary Materials

The following supporting information can be downloaded at: https://www.mdpi.com/article/10.3390/molecules29071568/s1, Figure S1: GC–MS chromatograms: (a) Sox extraction of NS hemp seeds. (b) Sox extraction of S hemp seed oil. (c) HPLC chromatogram of HSO (cold-pressed); Figure S2: Gene expression. mRNA levels of adipogenic gene (PPARγ and CEBPα) were investigated by quantitative RT-PCR after 72 h of culturing with CBD, THC, 0.05% HSO, or 0.1% HSO treatments without DM (a), or with DM (b); the mean ± SD are shown compared to their controls; ANOVA as * *p* ≤ 0.05, ** *p* ≤ 0.01, *** *p* ≤ 0.001; Table S1: Mean fold change (2^−ΔΔCt2^) of PPARγ and CEBPα mRNAs expression compared to the control (hMSC); Table S2: Mean fold change (2^−ΔΔCt2^) of PPARγ and CEBPα mRNAs expression compared to the control (DM-hMSC) and Table S3: Sequences of the primers used for qRT-PCR.
