# Supplementary material for: Hemp Seed Oil Inhibits the Adipogenicity of the Differentiation-Induced Human Mesenchymal Stem Cells through Suppressing the Cannabinoid Type 1 (CB1)
Source: Molecules. 2024 Mar 31;29(7):1568. doi: 10.3390/molecules29071568 (PMC11013118; doi:10.3390/molecules29071568)
Supplement: Supplementary file 1 [file molecules-29-01568-s001.zip › molecules-2895791-supplementary.pdf]

Supplementary Data

(a)

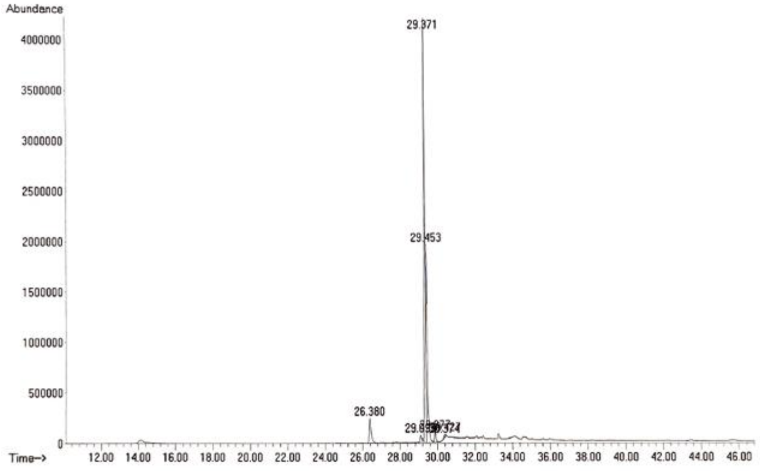

Sox, NS

(b)

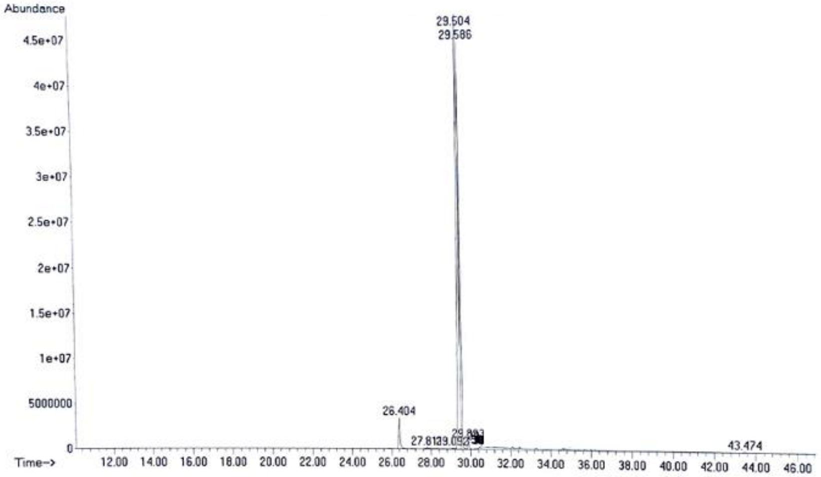

Sox, S

(c)

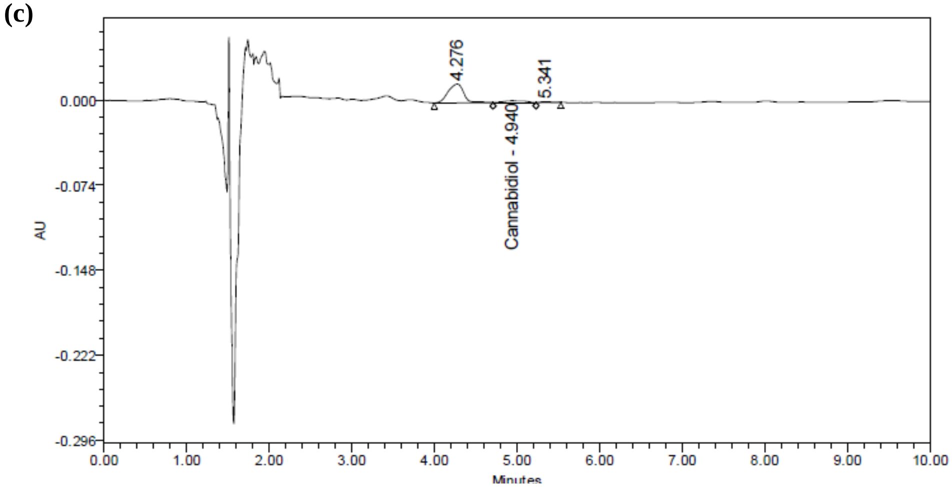

Supplementary Figure 1. GC-MS chromatograms: (a) Soxlet extraction of NS hemp seeds. (b) Soxlet extraction of S hemp seed oil. (c) HPLC chromatogram of HSO (cold pressed)

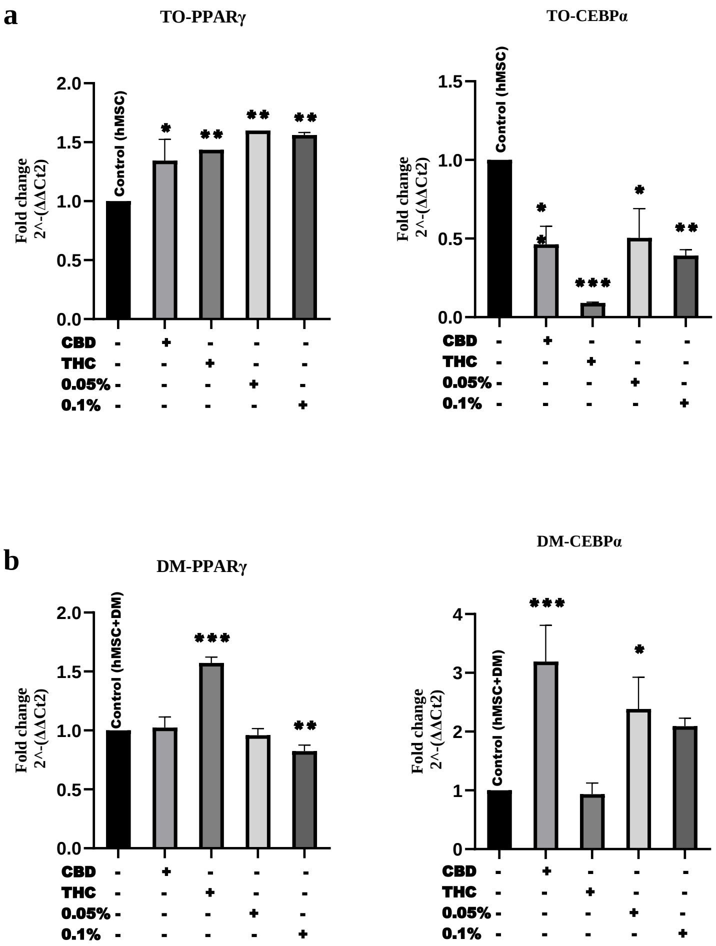

Supplementary Figure 2. Gene expression. mRNA levels of adipogenic gene (PPARγ and CEBPα) were investigated by quantitative RT-PCR after 72h of culturing with CBD, THC, 0.05% HSO, or 0.1% HSO treatments without DM (a), or with DM (b); the mean ± SD are shown compared to their controls; ANOVA as *p ≤0.05, **P ≤0.01, ***P ≤0.001.

Supplementary Table S1. Mean Fold change (2^-(ΔΔCt2)) of PPARγ and CEBPα mRNAs expression compared to the control (hMSC)

	**PPARγ mRNA mean of 2^-(ΔΔCt2)**	**CEBPα mRNA mean of 2^-(ΔΔCt2)**
**hMSC**	0	0
**TO-CBD**	0.345 ± 0.127	-0.538 ± 0.082
**TO-THC**	0.435 ± 0.113	-0.91 ± 0.004
**TO-0.05% HSO**	0.599 ± 0.023	-0.495 ± 0.131
**TO-0.1% HSO**	0.56 ± 0.016	-0.609 ± 0.027
**hMSC+DM**	26.153 ± 0.791	3.836 ± 0.797
**DM + CBD**	26.767 ± 0.902	14.078 ± 0.425
**DM + THC**	41.731 ± 2.191	3.413 ± 0.091
**DM + 0.05% HSO**	25.091 ± 1.846	10.233 ± 0.057
**DM + 0.1% HSO**	21.330 ± 0.375	9.039 ± 1.204

Supplementary Table S2. Mean Fold change (2^-(ΔΔCt2)) of PPARγ and CEBPα mRNAs expression compared to the control (DM-hMSC)

	**PPARγ mRNA mean of 2^-(ΔΔCt2)**	**CEBPα mRNA mean of 2^-(ΔΔCt2)**
**hMSC**	-0.963 ± 0.001	-0.787 ± 0.049
**TO-CBD**	-0.95 ± 0.008	-0.904 ± 0.002
**TO-THC**	-0.957 ± 0.002	-0.981 ± 0.006
**TO-0.05% HSO**	-0.941 ± 0.003	-0.897 ± 0.015
**TO-0.1% HSO**	-0.942 ± 0.002	-0.915 ± 0.028
**DM- hMSC**	0	0
**DM + CBD**	0.025 ± 0.09	2.191 ± 0.619
**DM + THC**	0.573 ± 0.049	-0.065 ± 0.191
**DM + 0.05% HSO**	-0.04 ± 0.057	1.386 ± 0.539
**DM + 0.1% HSO**	-0.176 ± 0.053	1.092 ± 0.136

Supplementary Table S3. Sequences of the primers used for qRT-PCR

Primers	Sequence
**GAPDH-F**<br>**GAPDH-R**	5′-GAGTCCACTGGCGTCTTC-3′<br>5′-GGGGTGCTAAGCAGTTGGT-3′
**PPARγ-F**<br>**PPARγ-R**	5′-GACCAGAAGCCTGCATTTCTGC-3′<br>5′-CTGTGTCAACCATGGTCATTTCGTT-3′
**CEBPα-F**<br>**CEBPα-R**	5′-AGGAGGATGAAGCCAAGCAGCT-3′<br>5′-AGTGCGCGATCTGGAACTGCAG-3′
**CB1-F**<br>**CB1-R**	5′- GTTCTAGCGGACAACCAGCC-3′<br>5′- TCAATCTCTTTGCCCCTTCGC-3′
**CB2-F**<br>**CB2-R**	5′- ACTCAACAGGTGCTCTGAGTGG-3′<br>5′- CTTGTCTAGAAGGCTTTGGGTTGTG-3′
**TRPV1-F**<br>**TRPV1-R**	5′- TTCGAGTAGCAACCGCCTTC-3′<br>5′- CCCAGTGTGCAACCAGCTAGA-3′
**GPCR55-F**<br>**GPCR55-R**	5′- ATGACATCTCTCAGCCCTCTCAG-3′<br>5′- ATCAGCTCGTTGACACCGTC-3′
**FAAH-F**<br>**FAAH-R**	5′- GAGGACATGTTCCGCTTGGA -3′<br>5′- AAGAAGGGAACCAGCGTGTG -3′
**MGL-F**<br>**MGL-R**	5′- GCAAACGAGGATCCGCTGC-3′<br>5′- GGGAGGTCCTGGTAGGGAAT-3′
